# Treatment of Emergencies

**Published:** 1887-05-28

**Authors:** 


*(To be continued.)*

### TILL THE DOCTOR COMES.

[Inquiries and Communications for this column should be addressed, "Physician," care of the Editor of THE HOSPITAL.]

### XIX.—TREATMENT OF EMERGENCIES.

*Wounds.*—With regard to the washing of old wounds, it is advisable never to use sponges or anything but what can be immediately burnt; otherwise foul discharges may inadvertently be carried from one wound to another, setting up inflammation, erysipelas, and the like. Tow, old rag, or something of the same kind is to be preferred, and immediately after use it (with the dressings that have been removed) should be burnt. Too much care cannot be taken in washing the hands after dressing, and in the matter of cleanliness of every sort and description. Old ointments must never be allowed to accumulate round a wound, for after a time they decompose, irritate the sound skin, and cause a larger wound. It is sometimes difficult to remove them, when attention has not been paid to this point; but some sweet oil, well rubbed over the surface of the deposit with a little cotton-wool, will gradually dissolve it, and then the skin may be gently washed with soap and warm water. Marks of strapping are easily removed in the same way: turpentine and spirits of wine are sometimes advised, but they are much too irritating to young, tender skin. In removing the strapping from a wound, care is requisite, for if one end of the strapping is seized, and the strip pulled off right across the wound, it must inevitably, when it reaches the other side, pull the two edges of the wound apart, and reopen it. Instead of this, both ends of the strapping should be taken, one in each hand, and they should be gradually drawn towards each other, till they meet in the centre over the wound.

In treating a fresh wound, it must first be well washed, all dirt, sand, etc., and any splinters that may be in it being removed; the bleeding, if any, must then be stopped by appropriate measures, and the place dressed in the best way to promote its healing. Wounds vary so much in their nature that some subdivisions must be made before their treatment can be with sufficient clearness indicated.

1. *Clean cut.*—Made with a sharp instrument. This is the best kind of wound for healing quickly. The great object is to bring the edges accurately together with strapping. This is best done by taking a narrow strip of strapping and warming it, and by commencing to apply it at such a distance on one side of the wound that it may get a firm hold of the skin. On reaching the wound, draw its outer edge well up to the inner one, adapt them carefully with the finger, and, when adapted, take the strapping quickly across, and fix it at an equal distance on both sides of the wound; apply other strips in the same way, till the wound is covered or nearly so; and lastly, place two strips diagonally across these to keep the whole in place. As a rule, the strapping will require changing every other day; but a wound should not be disturbed oftener than is necessary. In many cases a piece of dry lint and a bandage over the strapping will keep the parts opposed, and the strapping from slipping. A piece of dry lint on a cut finger is preferable to strapping, as the movement of the part will prevent strapping from sticking accurately. On the face, where a scar is a matter of some importance, it will be necessary, if the wound is of any size, to have the edges accurately sewn together.

2. *Bruised cut.*—Made by a blunt instrument, often with much force. The edges are here bruised, and will not unite so readily as in the former case. If there is not much bruising, an attempt may still be made to get the edges to heal quickly by strapping them together. If this is considered unadvisable, dry lint or water dressing may be applied. Water dressings and lotions are not nearly so much used as formerly, and quite rightly so: for when taken off, the dressing is filthy and foul smelling. Dry applications are now more general. A very good dressing for these cases is made by first cutting a small piece of gutta-percha tissue, as long as and rather wider than the cut, placing it carefully over the wound, and bandaging a piece of dry lint over that. This will only require changing every second or third day. Wounds usually heal readily under it, and the gutta-percha tissue prevents the lint from sticking to the wound and drawing the edges apart when it is changed.

3. *Stabs and deep wounds.*—These must on no account be brought together with strapping, or the skin would heal, leaving a cavity beneath it unhealed. The discharge would collect, and an abscess eventually form, which would have to be opened by slitting up the healed skin. These wounds must be made to heal up from the bottom, and if the skin tends to heal over too soon, a piece of lint must be introduced between the edges to keep it open. The gutta-percha tissue and dry lint is a useful dressing for these cases also, but disinfecting lotions must often be used in the after stages. Remember in these cases that an artery or deep vein may be injured, and ascertain the amount of bleeding before determining to treat it without surgical assistance.

4. *Lacerated wounds.*—These are large torn wounds, which will almost always require stitches and skilled treatment. Bleeding must be stopped, and any injury prevented to the wound till a doctor can arrive.

5. *Still larger wounds, as torn-off limbs.*—The same remarks will apply. Attend to the bleeding and give stimulants. Fortunately, torn vessels seldom bleed much, so that these wounds are not necessarily so immediately dangerous as would at first appear.

Any wound of a joint is very dangerous: it almost always results in a stiff joint, or perhaps worse. Always send for a surgeon immediately in such cases.

So much as to the immediate treatment of wounds. and providing the case goes on well, the same sort of dressing may be continued. But if the wound becomes hot and painful, if the edges look red and angry, if it seems to the patient to throb and shoot, then inflammation has set in: it is useless to look for immediate union; hot fomentations or poultices must be applied, and, if the wound is of any size, skilled advice must be sought.

